# Current metabolic perspective on malnutrition in obesity: towards more subgroup-based nutritional approaches?

**DOI:** 10.1017/S0029665120000117

**Published:** 2020-08

**Authors:** Ellen E. Blaak

**Affiliations:** Department of Human Biology, NUTRIM, School for Nutrition and Translational Research in Metabolism, Maastricht University, Maastricht, Netherlands

**Keywords:** Tissue-specific insulin resistance, Metabolomics, Lipidomics, Transcriptomics, Dietary intervention, Personalised approaches

## Abstract

Lifestyle intervention may be effective in reducing type 2 diabetes mellitus incidence and cardiometabolic risk. A more personalised nutritional approach based on an individual or subgroup-based metabolic profile may optimise intervention outcome. Whole body insulin resistance (IR) reflects defective insulin action in tissues such as muscle, liver, adipose tissue, gut and brain, which may precede the development of cardiometabolic diseases. IR may develop in different organs but the severity may vary between organs. Individuals with more pronounced hepatic IR have a distinct plasma metabolome and lipidome profile as compared with individuals with more pronounced muscle IR. Additionally, genes related to extracellular modelling were upregulated in abdominal subcutaneous adipose tissue in individuals with more pronounced hepatic IR, whilst genes related to inflammation as well as systemic low-grade inflammation were upregulated in individuals with primarily muscle IR. There are indications that these distinct IR phenotypes may also respond differentially to dietary macronutrient composition. Besides metabolic phenotype, microbial phenotype may be of importance in personalising the response to diet. In particular fibres or fibre mixtures, leading to a high distal acetate and SCFA production may have more pronounced effects on metabolic health. Notably, individuals with prediabetes may have a reduced response to diet-induced microbiota modulation with respect to host insulin sensitivity and metabolic health outcomes. Overall, we need more research to relate metabolic subphenotypes to intervention outcomes to define more optimal diets for individuals with or predisposed to chronic metabolic diseases.

Worldwide, the prevalence of obesity, insulin resistance (IR) and type 2 diabetes mellitus has grown dramatically since the 1980s (http://www.who.int/news-room/fact-sheets/detail/obesity-and-overweight). Driven by easy access to energy-dense foods and a sedentary lifestyle, obesity has become a major global health and socio-economic problem in the 21st century^(1)^.

Malnutrition may both be a cause as well as a consequence of overweight and obesity and refers to an abnormal physiological condition caused by inadequate, unbalanced or excessive consumption of macronutrients or micronutrients (http://wwwfaoorg/3/a-i4646epdf). Many low and middle-income countries are facing currently the double burden of disease where infection and undernutrition may occur simultaneously with a rapid increase in overweight and obesity, particularly in urban settings. It is not uncommon that overweight and undernutrition co-exist in populations, households or even individuals (http://www.who.int/news-room/fact-sheets/detail/obesity-and-overweight). Malnutrition in obesity may relate to a greater availability of inexpensive nutrient deficient foods, potentially inducing micronutrient deficiencies and a positive energy balance^(2)^. Furthermore, the increased adipose tissue mass may sequester vitamins, leading to reduced circulating concentrations and a reduced supply of these nutrients to other tissues. For example, vitamin D deficiency, i.e. reduced circulating concentrations of 25-hydroxyvitamin D_3_, has been associated with obesity, IR and type 2 diabetes mellitus^(3)^. Overall, a poor or altered intake of macro- or micronutrients in obesity or altered metabolism and pharmacokinetics in the obese individual may lead to an inadequate nutritional status. Additionally, treatment of extreme obesity involving bariatric surgery may sometimes worsen these nutrient deficiencies^(2)^.

One of the biggest challenges in the management of obesity and related complications is weight maintenance after successful weight loss. Weight regain may be partly driven by biological factors related to adipose tissue biology^(4)^ or related to a reduced RMR, adjusted for lean body mass^(5)^. A meta-analysis of weight loss studies in the USA, including twenty-nine studies with hypoenergetic diets (with or without exercise) with long-term (≥2 years) follow-up, showed that on average more than half the weight loss is regained after 2 years and more than three-quarters is regained after 5 years^(6)^. This poses the question whether there should be more focus on the adoption of a healthy lifestyle and not on weight loss *per se*. Notably, it has been shown that moderate 5 % weight loss already improves metabolic function in multiple organs simultaneously^(7)^. Additionally, lifestyle intervention in prediabetic individuals may reduce diabetes and metabolic syndrome risk by more than 50 % in different settings worldwide despite moderate weight loss^(8–10)^. These lifestyle programmes were based on the general guidelines for a healthy nutrition and physical activity but tailored towards the individual lifestyle. Nevertheless, within these tailored lifestyle interventions, 30 % of the participants do not respond or adhere to the intervention. The current paper focuses on whether a personalisation based on a metabolic or microbial phenotype may be effective to improve the success of nutritional intervention with respect to metabolic health.

## The concept of tissue-specific insulin resistance

Insulin plays a central role in nutrient partitioning within the body. Whole body IR reflects defective insulin action in tissues such as muscle, liver, adipose tissue, gut and brain, which may precede the development of cardiometabolic diseases^(11)^. IR may develop in different organs but the severity may vary between organs. Indeed, in the different prediabetic phenotypes the state of impaired glucose tolerance may be characterised by more pronounced peripheral or muscle IR, whilst the state of impaired fasting glucose by more pronounced hepatic IR^(12)^. Consistent with this, we previously showed that skeletal muscle lipid handling was disturbed in the impaired glucose tolerance state, characterised by an impaired postprandial insulin sensitivity, an increased postprandial TAG extraction and a reduced muscle lipid turnover, compared with individuals with impaired fasting glucose^(13,14)^.

The question arises whether the severity of IR at the tissue level may determine the response to dietary intervention. A first question that has to be addressed then is whether these phenotypes are really distinct. We addressed this question by characterising the plasma metabolome^(15)^ and lipidome^(16)^ and adipose tissue transcriptome profiles^(17)^ in individuals with either more pronounced hepatic IR or more pronounced muscle IR in the European multicentre DIOGenes trial in overweight and obese individuals^(18)^; and used the Cohort on Diabetes and Atherosclerosis Maastricht^(19)^ and the Maastricht study, a large population-based cohort in the Maastricht area^(20)^ as validation cohorts. Based on a 5 or 7-points oral glucose tolerance test with insulin and glucose concentrations we estimated hepatic IR and skeletal muscle insulin sensitivity indices (HIRI and MISI, respectively) as previously described and validated against a hyperinsulinemic clamp by Abdul Ghani *et al*.^(21)^, and optimised for the MISI calculation by means of cubic splining by O'Donovan *et al*.^(22)^. For descriptive purposes, we divided individuals in having no IR, having muscle IR (the lowest tertile of MISI), having hepatic IR (in the highest tertile of HIRI) or having both MISI and HIRI.

In general, overweight or obese women have a lower HIRI as compared to males^(15–17)^. This is in line with studies in obese men and women using hyperinsulinemic euglycemic clamp techniques^(23)^. Individuals with HIRI or combined HIRI/MISI in general have higher plasma TAG and a larger waist^(15–17)^.

## Tissue-specific insulin resistance and metabolome profiles

The characterisation of the metabolic profile received renewed attention by the availability of new metabolomics techniques. In previous studies higher levels of branched-chain amino acids, lactate, acylcarnitines, glycolytic intermediates, long-chain fatty acids as well as reduced concentrations of ketone bodies, lysophosphatidylcholines and tricarboxylic cycle intermediates are associated with the development of IR and (pre)diabetes^(16,24–26)^. We investigated whether in individuals with overweight or obesity but without diabetes, hepatic IR and muscle insulin sensitivity were characterised by distinct metabolic profiles. Metabolome profiles in DIOGenes were quantified by NMR metabolomics and results of DIOGenes were validated in the Maastricht study^(15)^. Hepatic, but not muscle IR, was associated with lower ketone body concentrations (acetoacetate and β-hydroxybutyrate) and lower concentrations of the ketogenic amino acids leucine and tyrosine, indicating reduced ketogenesis and β-oxidation in the liver with progressing IR. Both muscle and liver IR showed a blood metabolome profile of elevated (branched chain) amino acids, lactate and TAG and lower glycine concentrations^(15)^. Our findings showed that muscle and liver IR are characterised by partly distinct and partly overlapping metabolome profiles.

## Tissue-specific insulin resistance and lipidome profiles

Subsequently, we analysed the lipidome profiles (by liquid chromatography–MS-lipidomics) in tissue-specific IR phenotypes in the DIOGenes study^(16)^. We found that muscle IR was associated with higher circulating lysophophatidyl choline (LPC) concentrations, in particular of the LPC 18:2 and 18:1 species. This positive association was independent of body composition and body fat distribution. Several previous studies have shown associations of LPC and general indices of insulin sensitivity^(27)^. Also, *in vitro* studies in male myotubes showed that extracellular LPC (16:0) and LPC (18:1) can act as lipid signalling molecules and may activate peroxisome proliferator activated receptor-δ-dependent gene expression and reduce lipid-induced inflammation and IR^(28)^. Our data extend previous findings indicating that LPC may be specifically related to muscle insulin sensitivity.

In women, but not in men, hepatic IR was positively associated with the sum of plasma TAG and diacylglycerol. Importantly, these observed differences between men and women were unlikely to result from the known differences in body composition since adjustments for body composition and body fat distribution did not affect the results. Furthermore, the women in our study had less hepatic IR, lower plasma TAG and higher HDL concentrations as compared to men. In general, healthy premenopausal women have a higher capacity for fat storage without inducing hazardous cardiometabolic health risks^(29)^, also indicated as female advantage. Our findings of a worsening of blood lipid profile in women with the progression of hepatic IR are intriguing and might imply that women with hepatic IR ‘catch-up’ with men with regard to CVD risk and that the relationship between (hepatic) IR and cardiometabolic risk may be sex-dependent. In line, Kim and Reaven^(30)^ showed that the female advantage may not be explained by differences in insulin action *per se* but from an attenuation of the relationship between IR and cardiovascular risk, in particular in younger individuals.

The mechanisms behind this intriguing sexual dimorphism in hepatic lipid metabolism and IR remain to be determined. Notably, in our study most women were most likely in a premenopausal status. One potential mechanism may lie in the impact of oestrogens on hepatic lipid metabolism^(29)^ or in sex-related differences in insulin-mediated VLDL-TAG metabolism or *de novo* lipogenesis^(29,31)^.

## Tissue-specific insulin resistance and adipose tissue transcriptome profiles

The IR state is accompanied by an increased adipose tissue mass and adipose tissue dysfunction, characterised by an impaired lipid buffering capacity, and infiltration of immune cells. Both systemic lipid overflow as well as low grade inflammation have been related to the development of IR of non-adipose tissues such as the liver and skeletal muscle^(11)^. Previous studies investigating the subcutaneous adipose tissue (ScAT) transcriptome have found an upregulation of ScAT inflammatory pathways and a downregulation of lipid metabolism pathways and/or mitochondrial respiratory pathways in individuals with whole body IR *v.* BMI-matched controls^(32)^ or in obese women with IR as compared to lean insulin sensitive women^(33)^. To investigate whether differential ScAT transcriptome profiles were related to more pronounced muscle or hepatic IR, we analysed baseline RNA sequencing profiles in 368 individuals in overweight and obese individuals of the DIOGenes study and we subsequently validated mechanisms related to the systemic inflammatory profile in two independent cohorts Cohort on Diabetes and Atherosclerosis Maastricht and the Maastricht study^(17)^.

We showed that an altered extracellular matrix remodelling gene expression profile in abdominal ScAT was present in overweight and obese individuals with pronounced hepatic IR. Interestingly, an inflammatory gene expression profile was particularly present in individuals with primarily muscle IR. Based on the gene expression results, we proposed that an increased systemic inflammatory profile is a mechanism linking the increased expression of inflammatory genes in abdominal ScAT to muscle IR. In line with this, in the Cohort on Diabetes and Atherosclerosis Maastricht and the Maastricht study, an increased systemic low-grade inflammation profile, as reflected by the combined score of plasma markers of low-grade inflammation, was specifically related to muscle IR and not to liver IR. Although the relationship between adipose tissue inflammation and IR has previously been posed, the findings in the present study extend these previous unpublished results by indicating the tissue-specific nature of this relationship.

**Fig. 1.**
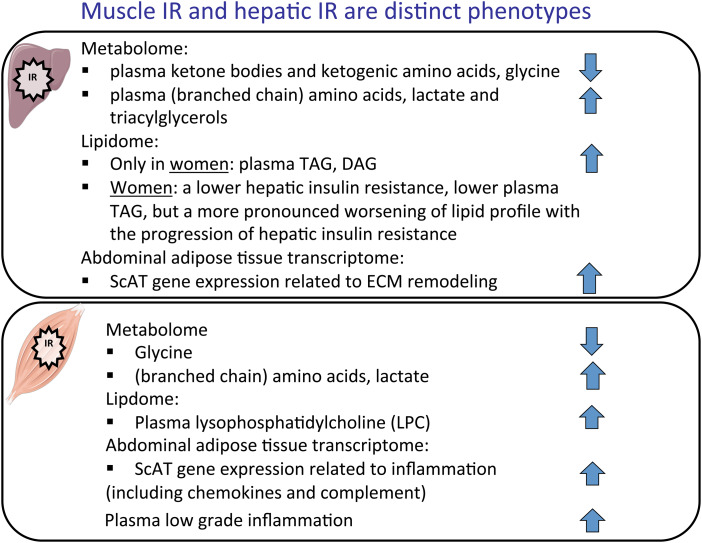
(Colour online) Metabolome, lipidome and adipose tissue transcriptome profiles in hepatic and muscle insulin resistance (IR). Upwards arrow indicates a positive association between hepatic or muscle IR, downwards arrow a negative association. Linear mixed model (Diogenes) or regression (Maastricht study) analyses with metabolite/lipid/gene expression as a dependent variable, hepatic insulin resistance indices/muscle insulin sensitivity indices as an independent variable, adjusted for BMI, waist:hip ratio and study centre included as a random effect (Diogenes), based on^(15–17)^. ScAT, subcutaneous adipose tissue; ECM, extracellular matrix.

Overall, the above results on metabolome, lipidome and transcriptome profiles indicate distinct tissue-specific IR phenotypes. The main characteristics of both phenotypes are summarised in Fig. 1.

## Tissue-specific insulin resistance: determinant of intervention response?

From the above it is evident the muscle and liver insulin resistant phenotypes are distinct which subsequently leads to the question whether these phenotypes respond differentially to dietary intervention. The above reported and previously shown disturbances in muscle lipid turnover in individuals with impaired glucose tolerance as compared to individuals with impaired fasting glucose were shown after ingestion of a high fat, high SFA mixed meal^(13,14)^. There are indications that dietary fat quality may impact muscle lipid handling. The first mechanistic evidence came from myotube studies showing that SFA preferentially accumulate as diacylglycerol, whilst unsaturated fatty acids are readily converted to TAG^(34)^. Also, a reduced fat oxidation was observed when diabetic myotubes were exposed to palmitic acid, whilst no differences were observed with oleic acid^(35)^. Indeed, we previously showed that under *in vivo* conditions the insulin resistant muscle is characterised by a reduced muscle lipid turnover as well as an increased muscle TAG extraction in particular after a high SFA-mixed meal and not after a PUFA meal^(36)^. Replacement of SFA by PUFA therefore would be protective against the development of peripheral IR. Manipulation of dietary fat quality by increasing the MUFA content or the *n*-3 or *n*-6 long-chain PUFA content of the diet in intervention studies has not shown consistent results on insulin sensitivity^(37–39)^. Based on the finding of differential fatty acid handling, dietary fat quality and peripheral insulin sensitivity, it can be speculated that the effectiveness of dietary fat manipulation may depend on initial metabolic tissue-specific phenotype. Indeed, a *post hoc* analysis in the European project LIGENE, focused on the study of dietary fat quantity and quality in the metabolic syndrome showed that insulin resistant metabolic syndrome individuals were more susceptible to a health effect from substitution of a high saturated fat diet by either high MUFA and high (complex) carbohydrate (with added *n*-3 PUFA) diets^(40)^. Unfortunately, in that study no distinction was made between liver and muscle IR.

Further evidence for an interaction between macronutrient composition and tissue-specific IR comes from a *post hoc* analysis of the CORDIO-PREV-DIAB study^(41)^. In the latter study, a low-fat high complex carbohydrate diet and the Mediterranean diet, rich in olive oil were compared with regard to outcomes related to glucose metabolism. A *post hoc* division in tissue-IR phenotype at baseline showed that individuals with muscle IR may benefit more from a Mediterranean diet showing a higher increase in disposition index (composite marker for insulin secretion adjusted for insulin secretion) as compared to the hepatic IR phenotype. Interestingly, individuals with hepatic IR have a more pronounced increase in disposition index on a low fat-high complex carbohydrate diet. Thus, although a diet according to existing dietary guidelines may be healthy for all, dietary prevention or treatment may require more personalised or sub-group based approaches to become most effective. Beside metabolic phenotype microbial phenotype may be of importance in personalising the response to diet. A landmark study within this field by Zeevi and coworkers^(42)^ showed that despite high interpersonal variability in postmeal glucose, personalised diets created with the help of a machine learning algorithm including dietary habits and physical activity and gut microbiota may successfully lower blood glucose responses. Personalisation based on microbial composition and phenotype has received increased attention, and in the following paragraphs this will be addressed targeting the microbial functionality with emphasis on the production of SCFA.

## Personalisation based on microbial phenotype

A growing body of evidence indicates an important role of gut microbiota and its functionality in the etiology of obesity, type 2 diabetes mellitus and CVD^(43)^. Our gut microbiota ferment indigestible dietary components that are incompletely hydrolysed by our digestive enzymes. Carbohydrate fermentation yields mainly SCFA as well as lactate and succinate and produces gases such as methane, carbon dioxide and hydrogen. Most carbohydrate fermentation takes place in the proximal colon and is ending in the transverse colon, The major SCFA, acetate, propionate and butyrate have been associated with positive effects on body weight control and insulin sensitivity^(44)^. Proteolytic fermentation, which occurs predominantly in the distal colon, yields a diverse range of metabolites: SCFA, gaseous products such as hydrogen, methane, carbon dioxide and hydrogen sulphide; the branched-chain fatty acids, isobutyrate, 2-methylbutyrate and isovalerate derived from fermentation of branched-chain amino acids and phenolic and indolic compounds derived from microbial fermentation of aromatic amino acids. Many of these compounds are toxic and have been considered as being detrimental for colonic and metabolic health (as reviewed in^43^).

Studies on the effects of SCFA in metabolic health are often hampered by the lack of information on actual SCFA kinetics including the rate of intestinal SCFA production, and subsequent absorption and eventual appearance in the circulation. Insight into the fate of SCFA and/or intestinal absorption will help to develop more targeted nutritional strategies. In a previous study, we addressed this by assessing SCFA release in the proximal (jejunum, ileum and proximal colon) intestines *v.* the distal intestines (descending colon, sigmoid and rectum) in patients undergoing abdominal surgery^(45)^. SCFA were highest in the inferior mesenteric vein and lowest in the radial artery (102 (se27) *v.* 22 (se8) μm). There was a 3-fold higher SCFA release from the distal intestine as compared to the proximal intestines. An implication of these findings is that slowly fermentable fibres that give lead to increased SCFA in the distal intestines may have a high potential to influence host metabolism.

Interestingly, we previously showed that acute distal colonic acetate administration increased circulating acetate concentrations and pronouncedly increased fasting fat oxidation (25 %), increased the concentration of satiety-stimulating hormone peptide YY and reduced concentration of the cytokine TNF-α in overweight males^(46)^. Acetate administration in the proximal colon did not affect the metabolic profile^(46)^. Subsequently, we showed that long-term supplementation with galacto-oligosaccharides, an acetogenic fibre, had no significant effect on peripheral and adipose tissue insulin sensitivity and no effects on faecal and circulating acetate and SCFA despite an increase in the abundance of bifidobacterium species^(47)^. In view of our findings that acute distal colonic acetate administration, but not proximal colonic administration affected parameters of metabolic health, we hypothesised that the site of fermentation of galacto-oligosaccharides may have led to an insufficient increase in systemic acetate to induce pronounced metabolic effects. Thus, increasing dietary fibre availability and SCFA formation in the distal colon may be an important determinant of metabolic health. In particular, since a higher distal carbohydrate fermentation may decrease detrimental proteolytic fermentation. This ‘microbial substrate switch’ might provide a novel dietary strategy for preventing and/or treating metabolic diseases^(43)^.

Based on these findings we hypothesised that combining a fast fermentable (to satisfy the proximal colonic microbiota) with a slow fermentable, acetogenic fibre will enhance distal acetate and SCFA production, thereby improving human substrate and energy metabolism. To test the hypothesis, we performed experiments in an *in vitro* model of the human colon (TIM-2), which was inoculated with faeces from lean and pre-diabetic overweight donors. At first, we selected the fibre combinations that led to the highest distal acetate production *in vitro* to subsequently execute an acute human intervention study with this mixture. In a first acute human study, we showed that a 1-d supplementation with a novel fibre combination of long-chain inulin and resistant starch led to pronounced effects on microbial fermentation as compared to supplementation with long-chain inulin only or placebo in lean but not in overweight prediabetic individuals the morning after the supplementation day. In lean individuals this was accompanied by an increased energy expenditure and carbohydrate oxidation and postprandial insulin sensitivity, whilst no metabolic effects were observed in the prediabetic individuals. Further mechanistic deepening on gut microbiota composition, SCFA in faeces and blood, as well as circulating hormones and metabolites will shed more light on these findings^(48)^. These data show that an innovative fibre mixture to target and reach the distal colonic microbiota might be a combination of readily fermentable oligomers and more complex fermentable fibres with a high degree of polymerisation and side chains. Nevertheless, our data show a lack of response in the prediabetic phenotype, which is consistent with previous findings showing that a 4-week oral butyrate administration altered metabolism and insulin sensitivity in lean but not in obese insulin resistant individuals^(49)^.

In the future, it is important to take the initial microbial composition and metabolic phenotype into account when targeting the microbiota and its functionality. Due to a putative resistance of the microbiome–host metabolism axis to dietary intervention in prediabetic individuals, intervention with fibre mixtures may require longer periods of time to become effective. Additionally, in future studies other factors that may shape the microbiota composition have to be taken into account such as sex, age, the use of medication and the gut transit time^(50–52)^. Of interest, we recently showed that long distal colonic transit, as measured with the radio-opaque methodology influences microbial diversification and fermentation in individuals with slow gastro-intestinal transit^(52)^.

## Conclusion

A diet based on existing guidelines for healthy nutrition may be a good diet for all. Nevertheless, these guidelines based on the reference person may not represent the optimal diet for all. Also, specific guidelines for the treatment (or prevention) of non-communicable diseases such as type 2 diabetes have been formulated ^(53,54)^. Overall, we need more research to relate metabolic subphenotypes to intervention outcomes to define more optimal diets for individuals with or predisposed to chronic metabolic diseases. In this, the different etiologies towards type 2 diabetes and cardiometabolic diseases have to be considered. Focusing on tissue-specific IR phenotypes as well as related microbial composition and functionality may be a targeted approach to define more personalised strategies for nutritional intervention. More prospective trials are required to provide the evidence for the implementation of these approaches.
